# Preeclamptic programming unevenly perturbs inflammatory and renal vasodilatory outcomes of endotoxemia in rat offspring: modulation by losartan and pioglitazone

**DOI:** 10.3389/fphar.2023.1140020

**Published:** 2023-04-25

**Authors:** Hagar A. Morgaan, Marwa Y. Sallam, Hanan M. El-Gowelli, Sahar M. El-Gowilly, Mahmoud M. El-Mas

**Affiliations:** ^1^ Department of Pharmacology and Toxicology, Faculty of Pharmacy, Alexandria University, Alexandria, Egypt; ^2^ Department of Pharmacology and Toxicology, College of Medicine, Health Sciences Center, Kuwait University, Kuwait City, Kuwait

**Keywords:** preeclampsia, endotoxemia, adult offspring, blood pressure, renal vasodilation, gestational therapy

## Abstract

**Introduction:** Preeclampsia (PE) enhances the vulnerability of adult offspring to serious illnesses. The current study investigated whether preeclamptic fetal programming impacts hemodynamic and renal vasodilatory disturbances in endotoxic adult offspring and whether these interactions are influenced by antenatal therapy with pioglitazone and/or losartan.

**Methods:** PE was induced by oral administration of L-NAME (50 mg/kg/day) for the last 7 days of pregnancy. Adult offspring was treated with lipopolysaccharides (LPS, 5 mg/kg) followed 4-h later by hemodynamic and renovascular studies.

**Results:** Tail-cuff measurements showed that LPS decreased systolic blood pressure (SBP) in male, but not female, offspring of PE dams. Moreover, PE or LPS reduced vasodilations elicited by acetylcholine (ACh, 0.01–7.29 nmol) or N-ethylcarboxamidoadenosine (NECA, 1.6–100 nmol) in perfused kidneys of male rats only. The latter effects disappeared in LPS/PE preparations, suggesting a postconditioning action for LPS against renal manifestation of PE. Likewise, elevations caused by LPS in serum creatinine and inflammatory cytokines (TNFα and IL-1β) as well as in renal protein expression of monocyte chemoattractant protein-1 (MCP-1) and AT1 receptors were attenuated by the dual PE/LPS challenge. Gestational pioglitazone or losartan reversed the attenuated ACh/NECA vasodilations in male rats but failed to modify LPS hypotension or inflammation. The combined gestational pioglitazone/losartan therapy improved ACh/NECA vasodilations and eliminated the rises in serum IL-1β and renal MCP-1 and AT1 receptor expressions.

**Conclusion:** Preeclamptic fetal programming of endotoxic hemodynamic and renal manifestations in adult offspring depends on animal sex and specific biological activity and are reprogrammed by antenatal pioglitazone/losartan therapy.

## 1 Introduction

Preeclampsia (PE) is a serious complication of pregnancy that affects 2%–8% of pregnancies and is considered a leading cause of morbidity and mortality. Epidemiological studies indicate that PE accounts for approximately 20% of all maternal deaths in low-income settings (McCarthy et al., 2018). PE is diagnosed by the presence of new onset hypertension >140/90 after 20 weeks of gestation associated with proteinuria and/or evidence of maternal acute kidney injury, liver impairment, and hematological and neurological signs (Brown et al., 2018). Offspring born to preeclamptic mothers are predisposed to elevated risks of cardiovascular disease, stroke, and mental disorders (Cheng and Sharma, 2016). Evidently, environment disruptions encountered during the fetal developmental phase play a seminal role in determining the offspring’s vulnerability to chronic disorders during later stages of childhood and adulthood (Peixoto et al., 2018).

PE management aims at stabilization of mothers and improve fetal maturity till labor. Commonly used therapies include antihypertensives (e.g., hydralazine, labetalol, or nifedipine), anticonvulsants (e.g., magnesium sulfate), and corticosteroids to promote the development of the fetal lung (Scott et al., 2022). Considering the pathogenic role of renin-angiotensin system (RAS) in PE progression (Irani and Xia, 2008; Siddiqui et al., 2010), pharmacologic RAS inhibition was thought to improve PE manifestations. Doering et al. (1998) and Zhou et al. (2008) showed that the PE-associated proteinuria, hypertension, and reduced pup weights in rodents are improved after angiotensin AT1 receptor blockade by losartan. The Peroxisome proliferator-activated receptor (PPAR-γ) negatively modulates RAS signaling and thereby favorably contributes to uteroplacental vascular and metabolic development (McCarthy et al., 2011b; Lane et al., 2019; Nesti et al., 2021). In fact, the blockade of PPAR-γ during gestation resulted in PE-like signs such as hypertension and endothelial dysfunction (McCarthy et al., 2011a). Moreover, PE complications are improved after treatment of PE dams with PPAR agonists like rosiglitazone or pioglitazone (McCarthy et al., 2011b; Allam and Masri, 2018; Lane et al., 2019).

Alternatively, sepsis is a life-threatening condition that results from exaggerated systemic immune and inflammatory responses to infections (Su et al., 2017). Systemic administration of LPS of the Gram-negative bacterial membrane is used to model the acute inflammatory response and tissue damage caused by sepsis (El-Mas et al., 2006; El-Lakany et al., 2018; Dickson and Lehmann, 2019). Clinical (Saia et al., 2015; Klein and Flanagan, 2016) and experimental studies (Losonczy et al., 2000) reveal that females are less vulnerable to immunological complications of endotoxemia than males. Likewise, we recently reported that endotoxic features of inflammation, hypotension, and cardiac autonomic neuropathy are demonstrated in male, but not age-matched female, rats (El-Lakany et al., 2018). Moreover, the exposure of rats to the LPS challenge causes sex-unrelated impairment in adenosinergically-mediated renovasclar vasodilations together with amplified mortality and renal inflammation in the male population (Wedn et al., 2020).

Considering the pivotal role of preeclamptic fetal programming in the escalated incidence of chronic illnesses in growing offspring (Cheng and Sharma, 2016; Peixoto et al., 2018), the current investigation determined whether the sexually-related inflammatory, hemodynamic, and renovascular vasodilator responsiveness to endotoxemia could be altered in the offspring of PE rats. Furthermore, given the positive modulation by PPARγ of renoprotection prompted by AT1 receptor blockade (Jin and Pan, 2007; Namikoshi et al., 2008; Lai et al., 2011), we asked if antenatal treatment with the combined pioglitazone/losartan therapy would be more influential than individual drugs in reprogramming the developed endotoxic defects.

## 2 Materials and methods

### 2.1 Animals

Adult Wistar rats (180–240 g) were used in the current study. Animals were obtained from the Animal facility of the Faculty of Pharmacy, Alexandria University, Egypt, and were maintained under controlled laboratory conditions and allowed free access to standard rat chow and tap water. All experimental protocols were approved by the Institutional Animal Care and Use Committee, Alexandria University, Egypt (Approval No. AU06201957149) and carried out in accordance with the Declaration of Helsinki and the Guide for the Care and Use of Laboratory Animals.

### 2.2 PE induction

For the induction of PE, N^ω^-nitro-L-arginine methyl ester (nitric oxide synthase inhibitor, L-NAME) (50 mg/kg/day) was administered via oral gavage for 7 consecutive days starting from day 14 of conception (Pandhi et al., 2001; Abuiessa et al., 2020). The measurements of SBP by the tail-cuff technique (see below) as well as urinary protein level were used to validate PE development.

### 2.3 The rat isolated perfused kidney

The isolated perfused kidney technique was carried out to assess renal vasodilator capacities to ACh and NECA according to the method described in previous studies (El-Mas et al., 2004; Gohar et al., 2014). The renal perfusion pressure was elevated by continuous infusion with the α_1_-adrenoceptor agonist phenylephrine (20 μM). Cumulative dose response curves to bolus injections of ACh (0.01–7.29 nmol) and NECA (1.6–100 nmol) were done by direct injection into the perfusate line proximal to the kidney.

### 2.4 Tail-cuff plethysmography

Non-invasive SBP measurements for conscious pregnant rats and adult offspring using the tail-cuff technique and a computerized data acquisition system with LabChart-7 pro software (Power Lab 4/30, model ML866/P, AD Instruments, Bella Vista, Australia) (El-Mas et al., 2015).

### 2.5 Immunohistochemistry

The method described in our previous studies (Helmy et al., 2015; Abuiessa et al., 2020) was employed to measure the expression of the inflammatory cytokine MCP-1 and Ang II AT1 receptors in glomerular tissues as well as in outer medullary areas of tubular cortex. Sections (4 μm thick) of kidney were deparaffinized in xylene and rehydrated in a series of declining ethanol concentration (100, 95% and 70%). Heat-induced epitope retrieval was carried out by immersing slides in coplin jars containing 10 mM citrate buffer solution and incubated in a microwave at power 100 for 1 min then power 30 for 9 min. Endogenous peroxidases were blocked by 0.3% hydrogen peroxide for 10 min. The rabbit, anti-rat primary antibodies, AT1 (1 μg/μL, Thermo Scientific^®^, Berlin, Germany and MCP-1 (1 μg/μL, Thermo Scientific^®^) were diluted (1:300), applied to the slides and then sections were incubated at 4°C overnight. The secondary antibody (HRP conjugate) was applied for 30 min. The chromogen 3, 3′-diaminobenzidine was prepared and applied as instructed by the manufacturer for protein visualization. Images of glomerular and tubular tissues were used to measure the percentage of chromogen 3, 3′-diaminobenzidine positive stained area in renal tissues.

### 2.6 Urine analyses

On gestational day 20, pregnant rats were kept in metabolic cages with mesh wire bottom made of stainless steel and allowed access to standard rat chow and water. The 24-h urine samples were collected under light mineral oil and stored at −80°C until processed (Abuiessa et al., 2020). Urinary protein levels were measured by the pyrogallol red method (Yalamati et al., 2016) using Cromatest standard kit (LiNEAR Chemicals, Spain) according to the manufacturer’s guidelines.

### 2.7 Serum analyses

Retro-orbital blood samples were withdrawn from thiopental (50 mg/kg i. p.) anesthetized rats prior to kidney isolation. The collected blood was permitted to coagulate for 15 min at room temperature then centrifuged at 1,200 *g* for 10 min. The aspirated serum was stored at −80°C for subsequent ELISA determination of IL-1β (Rat IL-1β Platinum ELISA Kit, eBioscience ™ BMS630, United States) and TNFα (Rat TNF alpha Platinum ELISA Kit, eBioscience ™ BMS622, United States) according to the manufacturer’s protocol. Creatinine was determined colorimetrically using commercially available kits (BioSystems, Spain).

### 2.8 Protocols and experimental groups

#### 2.8.1 Hemodynamic and renal effects of endotoxemia in offspring of PE rats

A total of 8 offspring rat groups, 4 males and 4 females (*n* = 7-8 each, 10 weeks old), were employed in this experiment to investigate the influence of endotoxemia on hemodynamic and renal profiles in offspring of PE and non-PE mothers. The 4 groups of each rat sex were assigned as (i) saline-treated non-PE offspring, (ii) LPS-treated non-PE offspring, (iii) saline-treated PE offspring, and (iv) LPS-treated PE offspring. Endotoxemia was induced in adult offspring by *i. p.* administration of a 5 mg/kg dose of LPS (Lv et al., 2006; Abuiessa et al., 2020). Four hours later, SBP was measured by the tail-cuff technique and rats were anesthetized with thiopental (50 mg/kg i. p). Blood samples were withdrawn from retro orbital plexus for serum analyses, left kidneys were isolated and perfused for the assessment of renal vasodilator to ACh and NECA as detailed above and right kidneys were collected for immunohistochemical determination of MCP-1 and AT1 receptor expression in renal tubular and glomerular tissues.

#### 2.8.2 Modulation by antenatal therapies of PE/LPS manifestations

This experiment investigated the effects of gestational administration of pioglitazone or losartan on hemodynamic and renal defects caused by PE or PE/LPS insult. Studies were performed in the male offspring only since the preceding experiment showed clearer and more significant PE/LPS interactions on renal vasodilations in male compared with female offspring. Losartan (10 mg/kg) and/or pioglitazone (5 mg/kg) were administered to pregnant rats along with L-NAME (50 mg/kg/day) for 7 consecutive days, starting from gestational day 14 till 20. Six groups of male offspring (n = 6-7 each, 10 weeks old) of PE mothers were used: (i) saline-treated male offspring of PE/losartan mothers, (ii) LPS-treated male offspring of PE/losartan mothers (iii) saline-treated male offspring of PE/pioglitazone mothers, (iv) LPS-treated male offspring of PE/pioglitazone mothers,(v) saline-treated male offspring of PE/losartan/pioglitazone mothers and (iv) LPS-treated male offspring of PE/losartan/pioglitazone mothers. LPS (5 mg/kg i. p.) was administered to all adult male offspring and 4 h later, rats were processed for measurement of SBP, blood collection, renovascular and immunohistochemical studies as described in the previous experiment.

### 2.9 Drugs

Lipopolysaccharide (LPS, from *E coli*, serotype 0111:B4), phenylephrine hydrochloride, 5 -N-ethylcarboxamidoadenosine (NECA, adenosine analogue), acetylcholine (ACh), *N*_ω_-Nitro-L-arginine methyl ester hydrochloride (L-NAME) (Sigma-Aldrich Co., St. Louis, MO, United States), losartan potassium, pioglitazone hydrochloride (PHARCO Pharmaceutical Co., Alexandria, Egypt), thiopental sodium (Biochemie, Vienna, Austria) and heparin (5000 IU/mL; Nile Pharmaceutical Co., Egypt) were purchased from commercial vendors. ACh and NECA were freshly prepared in distilled water and dimethyl sulfoxide (Loba Chemie Pvt Ltd, India), respectively. LPS, heparin and thiopental were dissolved in saline.

### 2.10 Statistics

Values are expressed as means ± S.E.M. The vasodilatory responses to ACh and NECA were expressed as the percentage of the precontraction level induced by 20 μM phenylephrine. The cumulative vasodilatory effects of ACh and NECA were computed by calculating the area under the curve (AUC) for individual experiments using trapezoidal integration and zero line as the baseline. In immunohistochemical studies, the percentages of stained areas were estimated. The one-way ANOVA followed by the Tukey’s *post hoc* test was used to assess statistical significance with probability levels <0.05.

## 3 Results

### 3.1 L-NAME provokes preeclamptic manifestations in pregnant dams

Daily injection of pregnant dams with L-NAME (50 mg/kg/day) for 7 consecutive days caused a significant rise in SBP compared with saline-treated (non-PE) group on gestational day 20 (138.5 ± 1.9 mmHg vs. 111.9 ± 1.3 mmHg). The 24-h urine samples collected on gestational day 20 showed a rise in urine protein levels in PE dams (282 ± 32 mg/dL vs. 89 ± 10 mg/dL) compared with control counterparts.

### 3.2 PE programming of hemodynamics and renal vasodilatory profiles in adult endotoxic offspring

**FIGURE 1:**
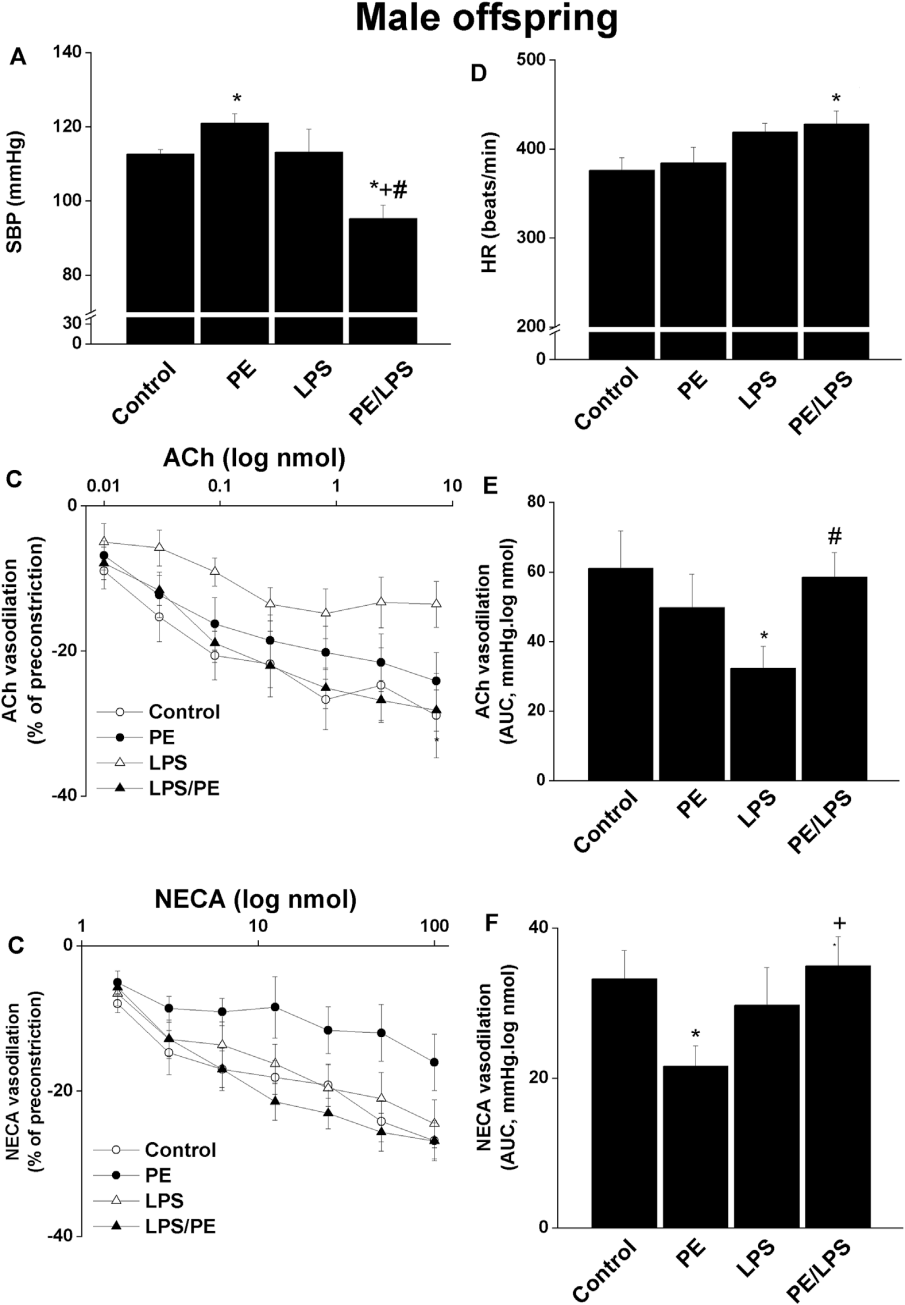
Effect of PE on tail cuff systolic blood pressure (SBP, panel **A**), heart rate (HR, panel **D**), cumulative vasodilatory dose response curves of ACh and NECA (panels **B, C**) and AUCs of the vasodilatory dose response curves (panel **E, F**) in phenylephrine preconstricted isolated perfused kidneys of endotoxic male offspring, respectively 4 h post intraperitoneal injection of LPS (5 mg/kg) or equal volume of saline. Data are expressed as the mean ± SEM of 7-8 measurements. ANOVA followed by the Tukey’s *post hoc* was utilized to measure statistical significance. *p < 0.05 vs. “Control”, ^+^ *p* < 0.05 vs. “PE” and ^#^ *p* < 0.05 vs. “LPS”.

The effects of PE on SBP, HR, and renal vasodilations caused by ACh (0.01–7.29 nmol) and NECA (1.6–100 nmol) in isolated perfused kidneys obtained from adult male offspring in absence and presence of endotoxic insult are shown in Figure 1. Compared to their respective non-PE group, a significant rise in SBP was observed in PE males (Figure 1A). Although the 4-h exposure of male offspring of non-PE dams to LPS (5 mg/kg, i. p) failed to modify SBP, the administration of the same LPS dose to male offspring of PE rats exhibited significant falls in SBP (Figure 1A) that were accompanied by acceleration of the heart (Figure 1D).

In male offspring, the renal vasodilatory responses to cumulative bolus injections of ACh (AUCs) in perfused kidneys were not affected by the PE insult but showed significant reductions in response to the 4-h LPS challenge (Figures 1B, E). To the contrary, the AUCs of NECA vasodilations were attenuated by PE and remained unaffected by LPS (Figures 1C,F). The individual suppressing actions of LPS or PE on ACh and NECA responses, respectively, disappeared in kidney preparations obtained from PE/LPS rats. In fact, the dose-dependent vasodilatory actions and AUCs for both vasodilators in the PE/LPS preparations were not statistically different from those of control non-PE, demonstrating a conditioning effect for the dual PE/LPS challenge against individual insults in male offspring (Figure 1).

**FIGURE 2:**
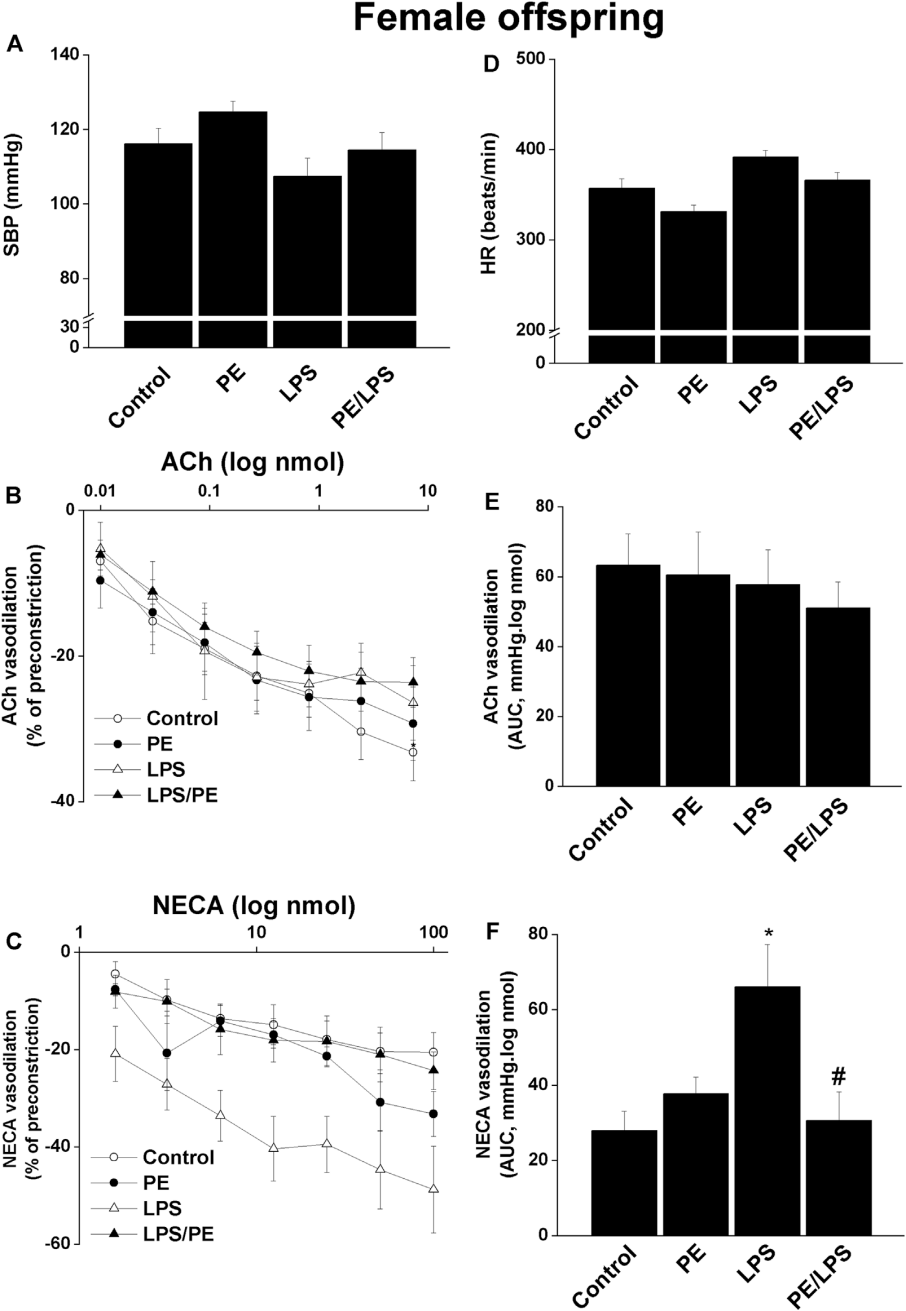
Effect of PE on tail cuff systolic blood pressure (SBP, panel **A**), heart rate (HR, panel **D**), cumulative vasodilatory dose response curves of ACh and NECA (panels **B, C**) and AUCs of the vasodilatory dose response curves (panel **E, F**) in phenylephrine preconstricted isolated perfused kidneys of endotoxic female offspring, respectively 4 h post intraperitoneal injection of LPS (5 mg/kg) or equal volume of saline. Data are expressed as the mean ± SEM of 7-8 measurements. ANOVA followed by the Tukey’s *post hoc* was utilized to measure statistical significance. *p < 0.05 vs. “Control”, ^#^ *p* < 0.05 vs. “LPS”.

Contrary to males, no significant changes in SBP (Figure 2A) or HR (Figure 2D) in response to PE and/or LPS insults were evident in female offspring. Moreover, the renal dose-response curves of ACh (Figure 2B) and AUCs (Figure 2E) were not influenced by PE programming, 4-h LPS exposure, or their combination. On the other hand, a facilitatory effect for LPS on NECA vasodilations was seen in renal preparations of female offspring obtained from non-PE, but not PE or PE/LPS, dams (Figures 2C,F).

### 3.3 Antenatal pioglitazone and losartan improve PE/LPS outcomes in male offspring

**FIGURE 3:**
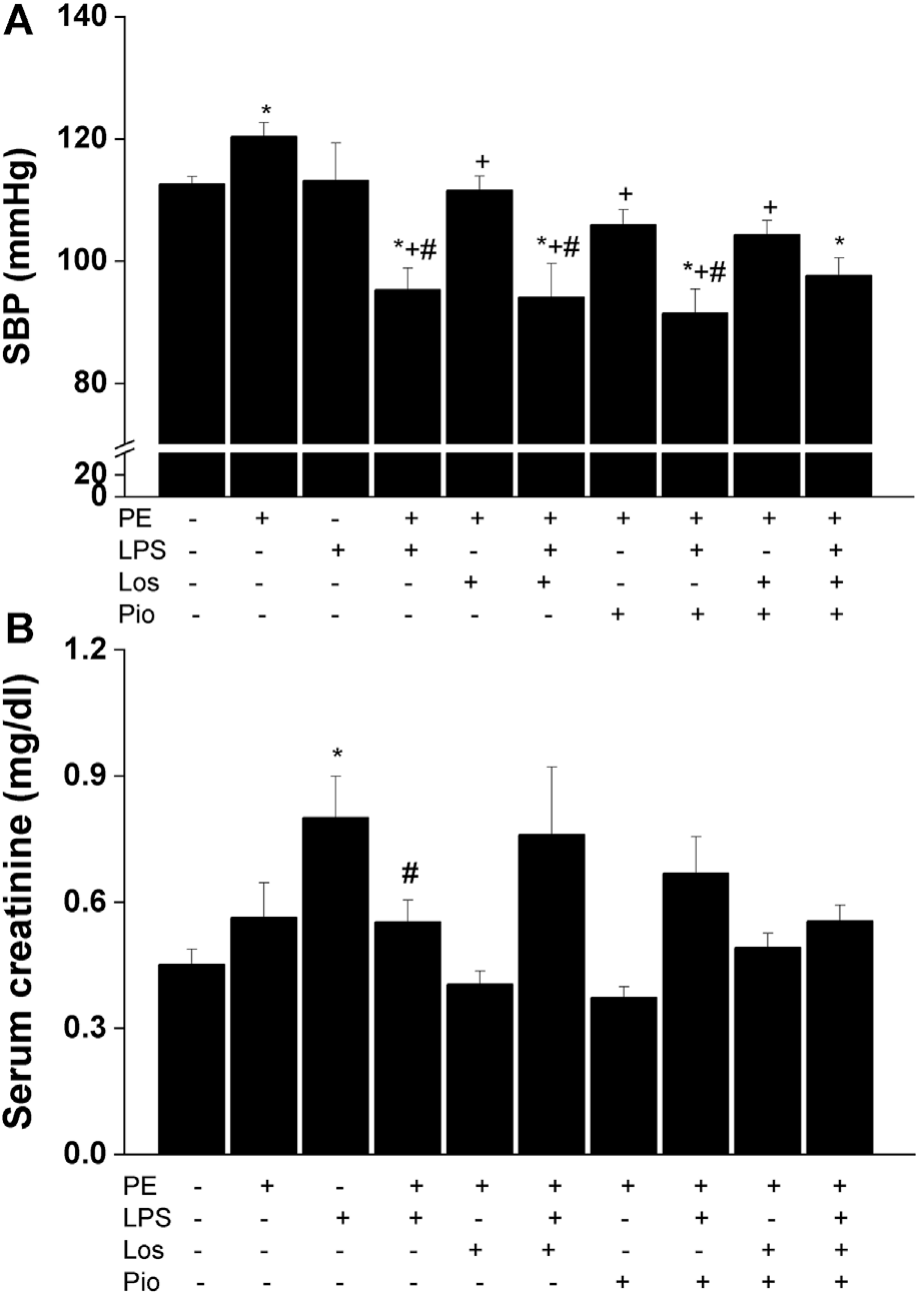
Effect of gestational losartan and/or pioglitazone therapy on SBP measurements (panel **A**) and serum creatinine (panel **B**) in male offspring of PE or PE/LPS dams. Values are expressed as means ± S.E.M of 7-8 measurements. The One-way ANOVA followed by the Tukey’s *post hoc* was utilized to measure statistical significance. *p < 0.05 vs. “Control”, ^+^ *p* < 0.05 vs. “PE” and ^#^ *p* < 0.05 vs. “LPS” values.

As noted earlier, this experiment was performed in the male offspring in which more significant PE/LPS interactions were observed. Urine analysis showed no statistical differences in urine protein levels between control (non-PE) and PE offspring (197 ± 13 vs. 234 ± 19 mg/dL). Moreover, similar levels of proteinuria were seen in PE rats treated gestationally with losartan, pioglitazone, or their combination (196 ± 25, 162 ± 16, and 205 ± 11 mg/dL, respectively). On the other hand, losartan, pioglitazone, or their combination abolished the elevation in SBP caused by PE and restored SBP to values like those found in the non-PE offspring (Figure 3A). However, none of these antenatal therapies affected the drop in SBP demonstrated 4 h post-LPS. Figure 3B illustrates an increase in serum creatinine in LPS-treated male offspring of non-PE rats and this effect was reversed when examined in their LPS-treated counterparts from PE dams. Likewise, no rises in serum creatinine occurred in male offspring of PE mothers receiving individual or combined antenatal therapies.

**FIGURE 4:**
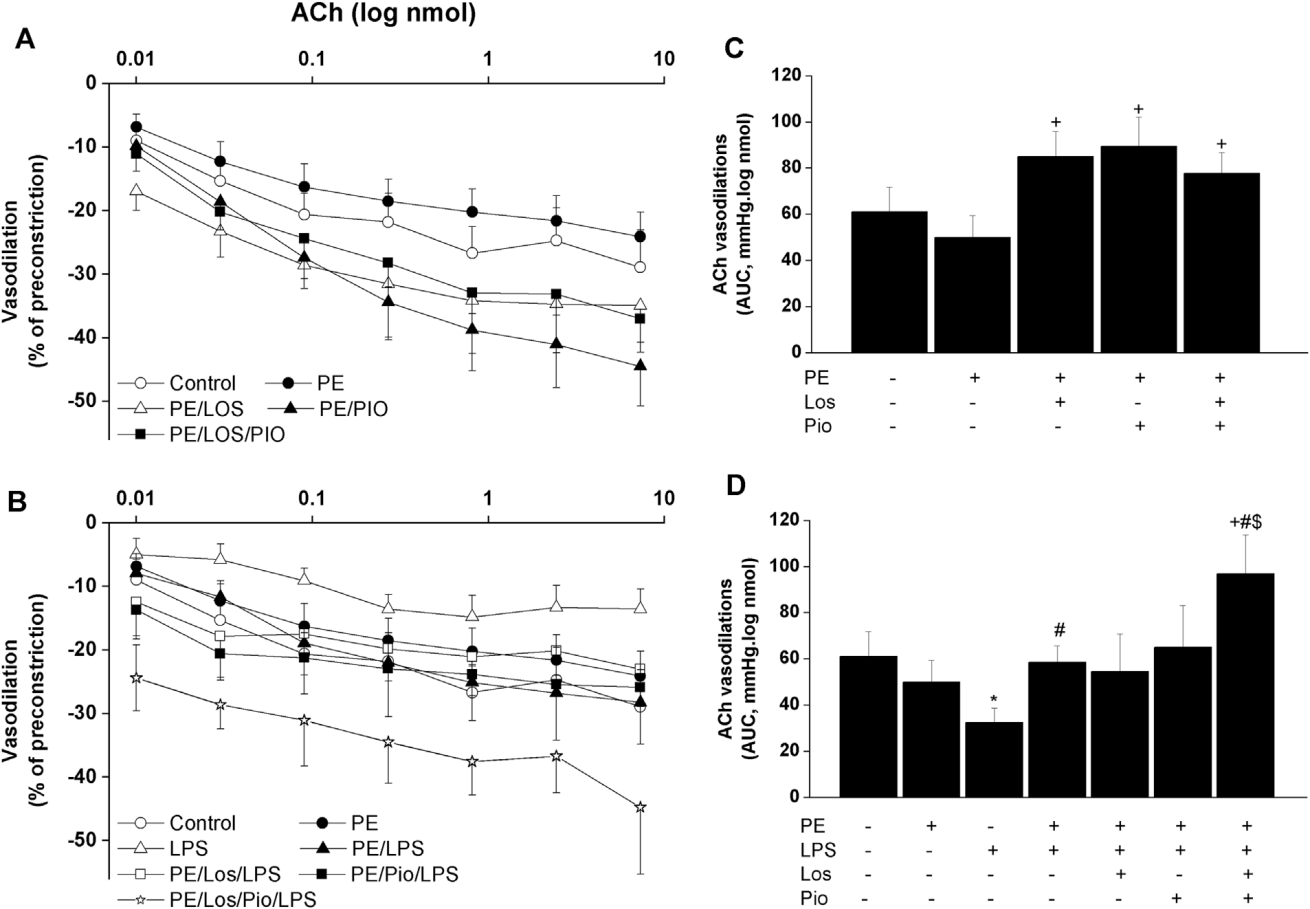
Effect of gestational losartan and/or pioglitazone therapy on cumulative vasodilatory dose response curves of ACh (panels **A, B**) and AUCs (panels **C, D**) of the ACh vasodilatory dose response curve in phenylephrine preconstricted isolated perfused kidneys in male offspring of PE or PE/LPS dams. Data are expressed as the mean ± SEM of 7-8 measurements. ANOVA followed by the Tukey’s *post hoc* was utilized to measure statistical significance. *p < 0.05 vs. “Control”, ^+^ *p* < 0.05 vs. “PE”, ^#^ *p* < 0.05 vs. “LPS” and ^$^ *p* < 0.05 vs. “PE/LPS” values.

**FIGURE 5:**
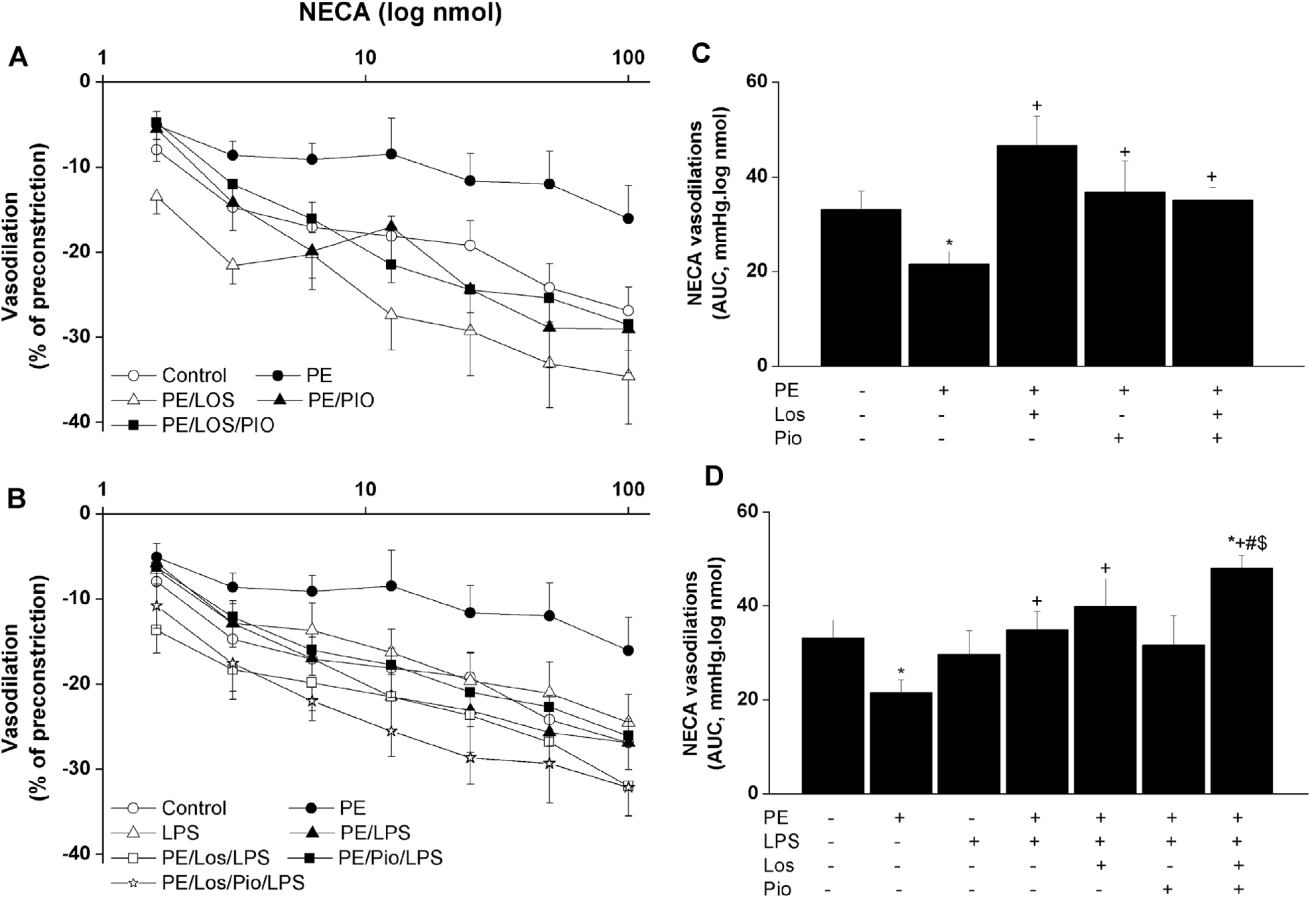
Effect gestational losartan and/or pioglitazone therapy on cumulative vasodilatory dose response curves of NECA (panels **A, B**) and AUCs (panels **C, D**) of the NECA vasodilatory dose response curve in phenylephrine preconstricted isolated perfused kidneys in male offspring of PE or PE/LPS dams. Data are expressed as the mean ± SEM of 7-8 measurements. ANOVA followed by the Tukey’s *post hoc* was utilized to measure statistical significance. *p < 0.05 vs. “Control”, ^+^ *p* < 0.05 vs. “PE”, ^#^ *p* < 0.05 vs. “LPS” and ^$^ *p* < 0.05 vs. “PE/LPS” values.

Renovascular studies showed that gestational administration of losartan, pioglitazone, or their combination increased ACh (Figure 4) and NECA (Figure 5) vasodilations in perfused kidneys obtained from PE dams. The amplified vasodilations were exemplified by the upward shifts in the dose-response curves of either vasodilator and significant rises in AUCs of the vasodilatory response (Figures 4, 5). The combined losartan/pioglitazone therapy caused more exaggerated rises in ACh/NECA responses in kidney preparations obtained from LPS-treated PE dams (Figures 4, 5).

### 3.4 Antenatal losartan and pioglitazone reverse the inflammatory response

**FIGURE 6:**
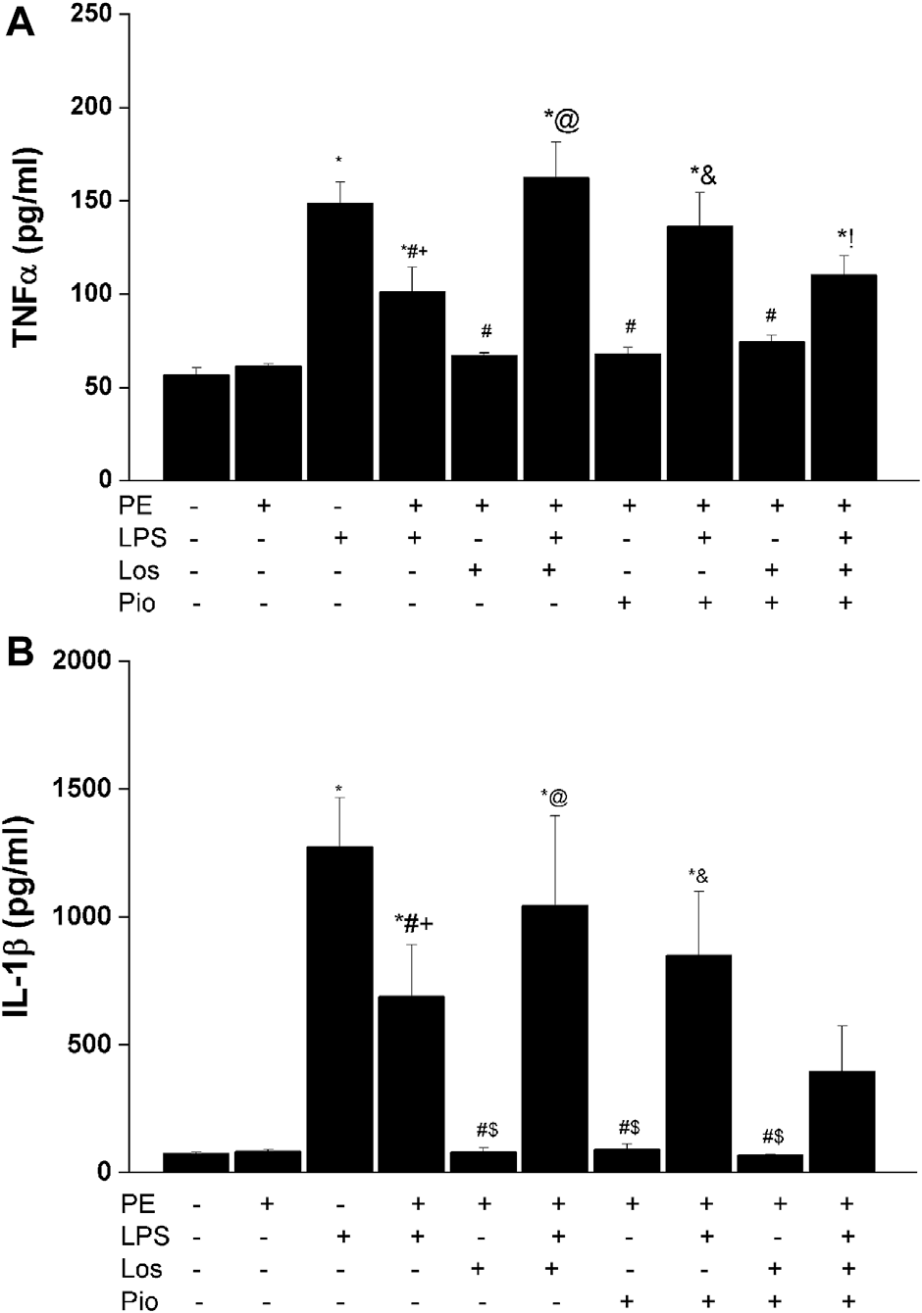
Effect of gestational losartan and/or pioglitazone therapy on serum levels of TNF-α (panel **A**) and IL-1β (panel **B**) in male offspring of PE or PE/LPS dams. Data are expressed as the mean ± SEM. The One-way ANOVA followed by the Tukey’s *post hoc* was utilized to measure statistical significance. *p < 0.05 vs. “Control”, ^+^ *p* < 0.05 vs. “PE”, ^#^ *p* < 0.05 vs. “LPS”, ^$^ *p* < 0.05 vs. PE/LPS, ^@^ *p* < 0.05 vs. “PE, Los” and ^&^ *p* < 0.05 vs. “PE, Pio” values.

**FIGURE 7:**
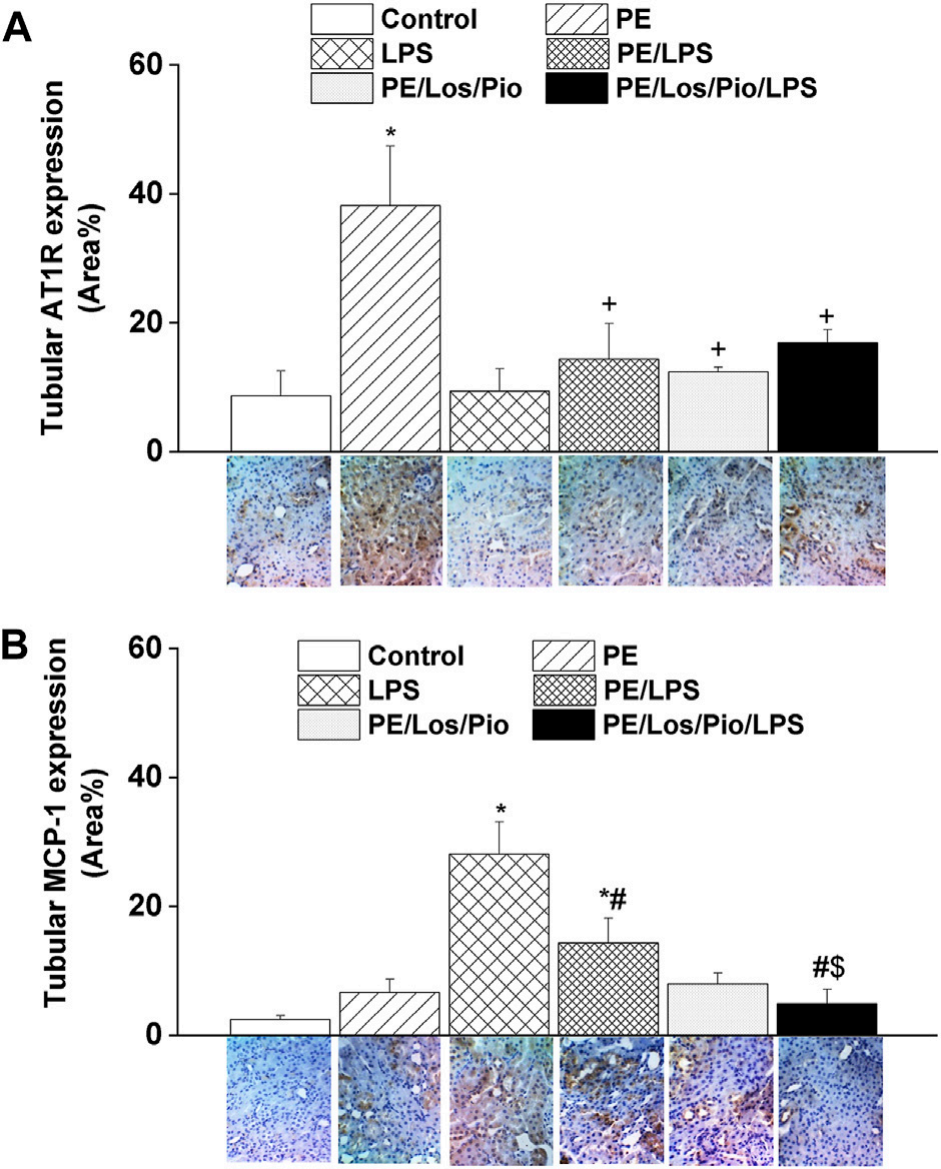
Effect of combined gestational losartan and pioglitazone therapy on immunohistochemical protein expressions of AT1 receptors (panel **A**) and MCP-1 (panel **B**) in renal tubular tissues of male offspring of PE or PE/LPS dams. The One-way ANOVA followed by the Tukey’s *post hoc* was utilized to measure statistical significance. Values are expressed as means ± S.E.M of 5 observations. *p < 0.05 vs. “Control”, ^#^ *p* < 0.05 vs. “LPS”, ^+^ *p* < 0.05 vs. “PE”, ^$^ *p* < 0.05 vs. “PE/LPS” values. Representative images for immunostained renal tubular tissues are shown below bar graphs.

**FIGURE 8:**
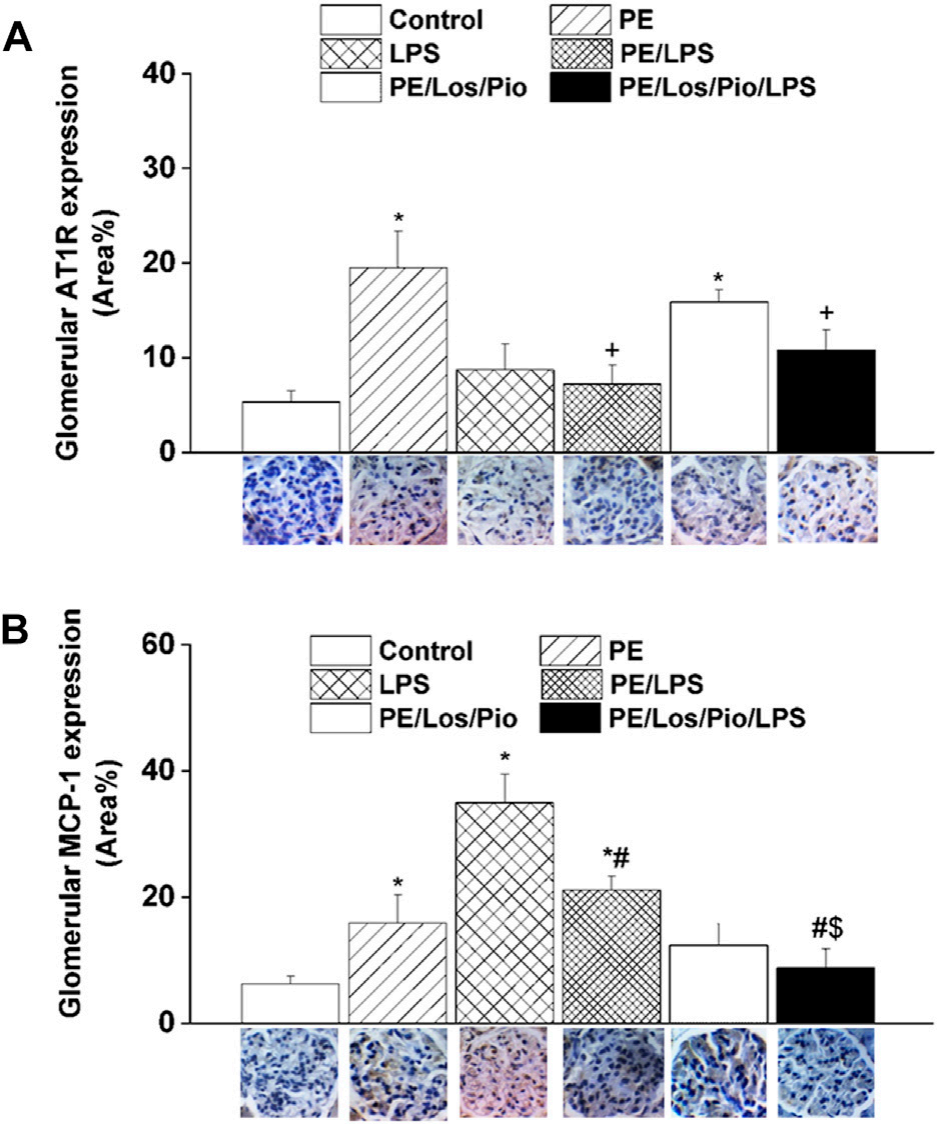
Effect of combined gestational losartan and pioglitazone therapy on immunohistochemical protein expressions of AT1 receptors (panel **A**) and MCP-1 (panel **B**) in renal glomerular tissues of male offspring of PE or PE/LPS dams. The One-way ANOVA followed by the Tukey’s *post hoc* was utilized to measure statistical significance. Values are expressed as means ± S.E.M of 5 observations. *p < 0.05 vs. “Control”, ^#^ *p* < 0.05 vs. “LPS”, ^+^ *p* < 0.05 vs. “PE”, ^$^ *p* < 0.05 vs. “PE/LPS” values. Representative images for immunostained renal glomerular tissues are shown below bar graphs.

Figures 6–8 depicts the effect of antenatal therapies on circulating and renal cytokines in male offspring. Compared with control rats, serum levels of TNF-α (Figure 6A) and IL-1β (Figure 6B) exhibited significant increases by 3- and 15-fold, respectively, in response to the endotoxic, but not to the PE, challenge. Moreover, immunohistochemical studies showed that the protein expression of renal tubular AT1 receptors (Figure 7A) and MCP-1 (Figure 7B) was increased by PE and LPS, respectively. Comparable changes in the expression of AT1 receptors and MCP-1 were observed in glomerular tissues (Figure 8). Such systemic/renal inflammatory signals incited by either insult were mostly and significantly attenuated in male offspring exposed to the double PE/LPS challenge (Figures 6–8). In all settings, the residual rises in serum IL-1β (Figure 6B) and tubular (Figure 7B) and glomerular (Figure 8B) MCP-1 expression were neutralized in male offspring of dams treated prenatally with the combined losartan/pioglitazone therapy.

## 4 Discussion

This study reports on the sex specificity of PE fetal programming of hemodynamic and renovascular sequels of endotoxemia in adult rat offspring and the reprogramming potential of prenatal pioglitazone/losartan therapy on these interactions. The data showed that while the female offspring of PE dams was minimally affected, the male progeny exhibited significant rises in SBP, which turned into a robust hypotension when challenged with LPS. Moreover, the renal vasodilatory responses to ACh/NECA were reduced by PE or LPS in male, but not female, offspring and these effects disappeared in PE/LPS preparations. Compared with individual therapies, the simultaneous prenatal exposure to losartan and pioglitazone caused significantly greater enhancement of renal vasodilations and suppression of biomarkers of circulating and renal inflammation in the male population. It is concluded that gestational losartan/pioglitazone boosts the conditioning effect of the dual PE/LPS challenge against individual insults caused by PE or LPS.

PE is a multifactorial gestational disease that is commonly characterized by hypertension, proteinuria and fetal growth retardation (Brown et al., 2018). Various experimental models have been developed to induce PE-like phenotype and imitate the pathological changes observed in humans. In the present study, daily injection of gravid dams with the NOS inhibitor L-NAME for 7 consecutive days elevated SBP and proteinuria compared with control counterparts. This is consistent with previous studies in which gestational NOS inhibition replicated some hallmarks of PE (Yallampalli and Garfield, 1993; Molnár et al., 1994; Chen et al., 2019). Remarkably, NO has been recognized as an important mediator involved in hemodynamic adaptation during gestation (Li et al., 2012).

The current findings favor the presumption that the female offspring are more resistance to hemodynamic and renal complications of endotoxemia and PE because the hypertensive response to PE as well as the reductions caused by LPS and PE in renal vasodilation caused by ACh and NECA, respectively, were all manifest in the male offspring only. These data are consistent with published data that immune, inflammatory, and cardiovascular anomalies caused by PE (Abuiessa et al., 2020; Birukov et al., 2020) or sepsis (Losonczy et al., 2000; Chen et al., 2014; El-Lakany et al., 2018) are sexually dimorphic and display more aggressive profiles in male offspring. The female sex hormones have been implicated, at least in part, in the presumed favorable profile in the female compared with the male population (Grigore et al., 2008; El-Lakany et al., 2018). Nonetheless, contradictory data of no sex differences or even worsened cardiovascular and metabolic outcomes in females than in males in the setting of PE (Habib et al., 2021; Beckers et al., 2022; Kuciene and Dulskiene, 2022; Ushida et al., 2022) and sepsis (Wedn et al., 2020; Fitzgerald et al., 2021) have been also recognized. Such discrepancies can be accounted for by differences in animal species, age, disease model, and specific biological activity under investigation.

In two recent studies from our laboratory (Abuiessa et al., 2020; Abuiessa et al., 2021), we provided the first experimental evidence that the exposure of weaning PE mothers or their male offspring to the endotoxic challenge intensifies cardiac autonomic neuropathy and upregulates the neuroinflammatory signal in the heart as well as in cardiovascular-sensitive nuclei of the brainstem. In the present study, we investigated whether the dual PE/LPS hit could elicit similar exaggerated damaging effects on renovascular reactivity in adult offspring. Contrary to our expectation, the present data in male offspring revealed advantageous sequels for the joint PE/LPS strategy on indices of renal function compared with individual offences. This view is supported by the observations that (i) the attenuated ACh or NECA renal vasodilations caused by LPS and PE, respectively, vanished in kidney preparations obtained from PE/LPS offspring, and (ii) the rises caused by LPS in serum creatinine, a biomarker of kidney injury, was eliminated in the PE-programmed offspring. It is tempting to speculate that the dual PE/LPS challenge defies renal damage caused by individual insults in male offspring. In this circumstance, while PE programming acts conceivably as a preconditioning stimulus that counterbalances the adverse renal response to consequent endotoxemia, LPS offers a postconditional opportunity that lessens the renal injurious reaction caused by prior PE programming. Remarkably, the phenomenon of pre- or post-insult conditioning has been described in a variety of pathological states. For instance, LPS was found to reduce infarct volume following middle cerebral artery occlusion (Sardari et al., 2020). Postconditioning with repeated mild hypoxia promotes brain rehabilitation and protects neonatal hypoxia-ischemic rats from brain damage (Deng et al., 2018). LPS preconditioning protects against organ damage following ischemic reperfusion in cardiorenal and brain tissues (Ha et al., 2008; Vartanian et al., 2011; He et al., 2018).

The question whether alterations in inflammation and chemotaxis could underlie the renoprotection conferred by the PE/LPS challenge against individual insults in the male offspring was investigated. The inflammatory response to LPS was validated by the substantial increments in circulating TNFα and IL-1β, two key proinflammatory cytokines that characterize the early immune response to endotoxemia (Lu et al., 2008; Dickson and Lehmann, 2019). Additionally, the upregulated protein expression of MCP-1, a chemoattractant protein (Singh et al., 2021), in renal tubular and glomerular tissues suggests an enhanced recruitment of monocytes and other immune cells into renal tissues in endotoxic rats. Moreover, MCP-1 provokes systemic inflammation following TLR-4 activation and is readily elevated in animal models of sepsis (Ramnath et al., 2008). Amazingly, these intensified signals of inflammatory and chemoattractant molecules of the endotoxic response were significantly attenuated in rats challenged with the dual PE/LPS insults, suggesting a quashing influence for prior PE programming on cytokine/chemokine responses to endotoxemia. By the same token, this may as well provide the molecular basis for the capacity of the combined PE/LPS maneuver to alleviate the compromised renal vasodilation caused by individual insults. In a similar situation, the preconditioning and neuroprotective actions of LPS against ischemic stroke injury has been attributed to the upregulation of antiinflammatory type I interferon-associated genes and suppression of NFκB activity (Vartanian et al., 2011). Others have correlated the LPS-induced tolerance against renal ischemic reperfusion insult to the activation of PPAR-γ and attenuation of oxidative stress or nitric oxide generation (Chatterjee et al., 2002; Collino et al., 2005).

Considering the key roles of RAS (Irani and Xia, 2008; Siddiqui et al., 2010) and its negative modulator PPAR-γ (McCarthy et al., 2011b; Lane et al., 2019; Nesti et al., 2021) in PE progression, one prime objective of the current study was to investigate if antenatal exposure to a combined regimen of the AT1 receptor antagonist losartan plus the PPAR-γ agonist pioglitazone would be more effective than separate therapies in reforming renovascular and inflammatory perturbations observed in the current model system. The rectifying effect of these pharmacologic therapies was verified by the ability of all three regimens to indiscriminately reverse the PE-associated suppression of renovascular dilations in male rats (Figure 5C). More relevantly, the combined losartan/pioglitazone regimen produced super-physiologic levels of renal vasodilations in PE/LPS rats that were well above increments caused by individual therapies (Figure 4D; Figure 5D). This heightened vasodilatory response was associated with, and possibly motivated by, an intensified antiinflammatory potential as reflected by the elimination of the residual rises in circulating IL-1β and renal MCP-1. Our findings are echoed by published clinical and experimental data that PPARγ activation enhances the renoprotective effect of AT1 receptor blockers in nephropathic states (Jin and Pan, 2007; Namikoshi et al., 2008; Lai et al., 2011). The negative modulation of RAS signaling (Lai et al., 2011) and oxidative and nitrative stresses (Kong et al., 2012) have been proposed as possible mechanisms for the enhanced renoprotection. Others have demonstrated that a regimen of losartan plus rosiglitazone exhibits additive renoprotection via proportional reductions in renal abundance of transforming growth factor-β and adhesion molecules (Lai et al., 2011).

The observation that gestational therapies failed to modify the hypotensive response to LPS in male offspring of PE dams despite the suppression of the concomitant inflammatory signal deserves a comment. Admittedly, the hypotensive response to endotoxemia is often set off by the developed state of systemic inflammation, which begins with the LPS-mediated upregulation of the TLR-4/NFκB/TNFα/iNOS cascade and consequent overproduction of NO, diminution of vascular resistance, and widespread systemic vasodilation (El-Mas et al., 2008; Singh et al., 2022). Since the inflammatory response was obliterated by gestational therapies particularly the combined losartan/pioglitazone regimen, it is likely that factors other than the inflammatory milieu might be responsible for the persistent fall in blood pressure. The upsurges in the renal vasodilatory propensity induced by individual, and more so by the combined, therapies may account, at least partly, for the maintained hypotensive response. The likelihood of this assumption gains credence from the reported intimate relationship between renal and vascular homeostasis (Kuczeriszka and Wąsowicz, 2022). Another possible mechanism may relate to renal AT1 receptors, whose expression was enhanced in PE offspring and restored to control levels after exposure to gestational losartan/pioglitazone therapy (Figure 7A). While the increased abundance of renal AT1 receptors is thought to contribute to the preeclamptic rise in blood pressure in this and other studies (Burke et al., 2016), the restoration of normal expression levels of the peptide receptors would help counterbalance the preeclamptic rises in blood pressure.

Despite the seemingly beneficial effects of targeting AT1 receptors (blockade) and PPAR-γ (activation) in the current study and those reported by others (Doering et al., 1998; Zhou et al., 2008; McCarthy et al., 2011b; Lane et al., 2019), caution should be taken when extrapolating the current experimental data to the human setting. Arguably, the gestational use of AT1 receptor blockers has often been discouraged because of their teratogenic potential. Fetal RAS blockade is associated with renal failure, fetal growth retardation, pulmonary hypoplasia and limb contractures (Alwan et al., 2005; Bullo et al., 2012). Further, little information is available regarding the safety of thiazolidinediones like pioglitazone during pregnancy (Kung and Henry, 2012). It should be noted, however, that the utilization of drugs such as losartan and pioglitazone as experimental tools could help us unveil important insight into the understanding of the mutual interaction between AT1 receptor and PPAR-γ pathways in PE pathophysiology and reveal possible therapeutic clues for reprogramming renal defects induced by PE in adult offspring.

In conclusion, the study provides novel data that highlight a protective effect for the dual PE/LPS challenge against adverse renal consequences evoked by individual insults in adult offspring. The suppressed renal vasodilatory activity as well as the preconditioning and postconditioning influences of PE and LPS, respectively, are sexually differentiated and appeared in the male offspring only. Gestational therapy of PE dams with the combined losartan/pioglitazone regimen are more superior than individual therapies in suppressing the LPS-mediated escalation of the inflammatory state and depression of renovascular reactivity.

## Data Availability

The original contributions presented in the study are included in the article/supplementary material further inquiries can be directed to the corresponding author.

## Abbreviations

ACh, Acetylcholine; AT1, Angiotensin II type 1 receptor; AUC, Area under the curve, 3, 3′-diaminobenzidine; HR, Heart rate; iNOS, Inducible nitric oxide synthase; i.p, intraperitoneal; IL-1β, Interleukin-1 beta; LPS, Lipopolysaccharides; MCP-1, Monocyte chemoattractant protein-1; NECA, N-ethylcarboxamidoadenosine; L-NAME, N^ω^-nitro-L-arginine methyl ester; PPAR-γ, Peroxisome proliferator-activated receptor; PE, Preeclampsia; RAS, Renin-angiotensin system; SBP, Systolic blood pressure; TNFα, Tumor necrosis factor-alpha.
